# Enhanced derivation of human pluripotent stem cell-derived cortical glutamatergic neurons by a small molecule

**DOI:** 10.1038/s41598-017-03519-w

**Published:** 2017-06-12

**Authors:** Shi-Ying Cao, Yao Hu, Cheng Chen, Fang Yuan, Min Xu, Qi Li, Kai-Heng Fang, Yaoyu Chen, Yan Liu

**Affiliations:** 1 Institute for Stem Cell and Neural Regeneration, School of Pharmacy, Nanjing Medical University, Nanjing, China; 2 Department of Student Affairs, Kangda college of Nanjing Medical University, Lianyungang, China

## Abstract

Human pluripotent stem cells (hPSCs) play important role in studying the function of human glutamatergic neurons and related disease pathogenesis. However, the current hPSC-derived cortical system produced a significant number of inhibitory GABAergic neurons that reduced the purity of excitatory neurons. In this study, we established a robust hPSC-derived cortical neurogenesis system by applying the SHH inhibitor cyclopamine. Cyclopamine specified the dorsal cortical fate in a dose-dependent manner and enhanced the generation of cortical glutamatergic neurons, expressing PAX6, TBR1, TBR2, CTIP2, SATB2, and vesicular glutamate transporters (vGLUT). In contrast, the ventral patterning was inhibited and the GABAergic neurons were significantly reduced to 12% with the treatment of cyclopamine. In addition, we applied our current method to generate trisomy 21 iPSC-derived glutamatergic neurons that showed a robust reduction of vesicular glutamate transporters in the glutamatergic neurons with trisomy 21, revealing the developmental deficits in cortical glutamatergic neurons. Our method enriched the generation of cortical glutamatergic neurons which may facilitate the study of human neurological diseases and cell therapy.

## Introduction

Cortical glutamatergic neuron originates from dorsal telencephalon and comprises the major excitatory network in the central nervous system^1^. Glutamatergic neurons play critical roles in controlling cognitive, emotion, language, and motor function^2, 3^. Dysfunction of cortical glutamatergic neurons may be relevant to neurological diseases, such as Alzheimer’s disease^4^ and Down syndrome^5, 6^. Availability of glutamatergic neurons *in vitro* may represent cell-based strategies for transplantation therapy and etiology studies.

Human pluripotent stem cells (hPSCs), including human embryonic stem cells (hESCs) and human induced pluripotent stem cells (iPSCs)^7, 8^, provided opportunities to generate a specific type of human cells to study the human diseases and regenerative medicine dynamically. We and other groups have generated region-specific neurons including cortical glutamatergic neuron from hPSCs^9–14^. In the advent of direct differentiation technology, the progress has been made in disease modeling by using patient iPSC-derived glutamatergic neurons^15–18^. However, the hPSC-derived glutamatergic neurons were mixed with numbers of GABAergic neurons^17–19^. The heterogeneity of hPSC-derived neurons impeded future application of hPSCs in cell therapy and disease modeling. Many efforts have been put to enhance the purity of hPSC-derived glutamatergic neurons.

GABAergic neurons originate from ventral telencephalon, which is adjacent to the dorsal telencephalon^20^. Thus, inhibition of ventral patterning may enrich the population of glutamatergic neurons that derived from hPSCs. Sonic hedgehog (SHH) plays a key role in regulating the development of ventral telencephalon^3, 21^. The SHH inhibitor cyclopamine was applied to efficient inhibit ventral patterning during neuronal differentiation from mouse embryonic stem cells^22, 23^. However, few studies reported significant ventral inhibition with the treatment of cyclopamine in human neural differentiation^24–26^.

In this study, we applied the SHH inhibitor cyclopamine to promote the differentiation of glutamatergic neurons from hPSCs. We showed that cyclopamine reduced the population of ventral GABAergic neurons, while enriched the cortical glutamatergic neurons significantly. Following this method, we applied cyclopamine to trisomy 21 iPSCs and the euploid iPSCs to generate glutamatergic neurons. Cyclopamine treated trisomy 21-glutamatergic neurons exhibited robust reduction of vesicular glutamate transporters in comparison to the treated euploid control, which were not observed in untreated excitatory neurons.

## Results

### hESC-derived telencephalic progenitors partially differentiated into ventral neurons by default differentiation

hPSCs differentiated into dorsal telencephalic progenitors and subsequently largely became glutamatergic neurons without additional morphogens^9, 10, 27–29^. By following our previous reported method (Supplementary Fig. S1a)^30^, we differentiated hESCs (H9) to the neural epithelial cells, expressing PAX6, SOX1, SOX2 and KI67 at day 10, as well as the telencephalic markers FOXG1 and OTX2 (Supplementary Fig. S1b). With further differentiation, hESC-derived neuroepithelial cells largely expressed the cortical transcription factor PAX6 at day 21 (Supplementary Fig. S2a). After day 27, dorsal telencephalic progenitors gradually expressed the cortical progenitor marker TBR2 (Supplementary Fig. S2b). The cortical deeper-layer markers TBR1 and CTIP2 were observed after day 40 (Supplementary Fig. S2b). The cortical upper-layer marker SATB2 was detected after day 125 (Supplementary Fig. S2b). Vesicular glutamate transporters (BNPI, also named vGLUT) were exclusively concentrated in glutamatergic nerve terminals to form asymmetric excitatory-type synapses over 100 days of differentiation (Supplementary Fig. S2d)^31, 32^. Thus, hESCs spontaneously differentiated into cortical glutamatergic neurons. However, we observed that dorsal PAX6^+^ progenitors were mixed with the lateral ganglionic eminence (LGE) progenitors that expressing MEIS2 (Supplementary Fig. S2a) or ventral cells that expressing ISLET1 (Supplementary Fig. S2c). Furthermore, numbers of GABA^+^ neurons co-expressed the LGE marker MEIS2 (Supplementary Fig. S2c). In addition, the expression of striatal projection neuronal marker DARPP32 and GAD67 (GABA synthesis enzyme) confirmed that the neurons were ventral cells (Supplementary Fig. S2c). These results suggested that ventral cells were also derived from hESCs by default differentiation.

Taken together, our results showed that hESCs-derived cortical glutamatergic neurons were mixed with numbers of ventral GABAergic neurons.

### Cyclopamine efficiently inhibited the ventral patterning

**Figure 1:**
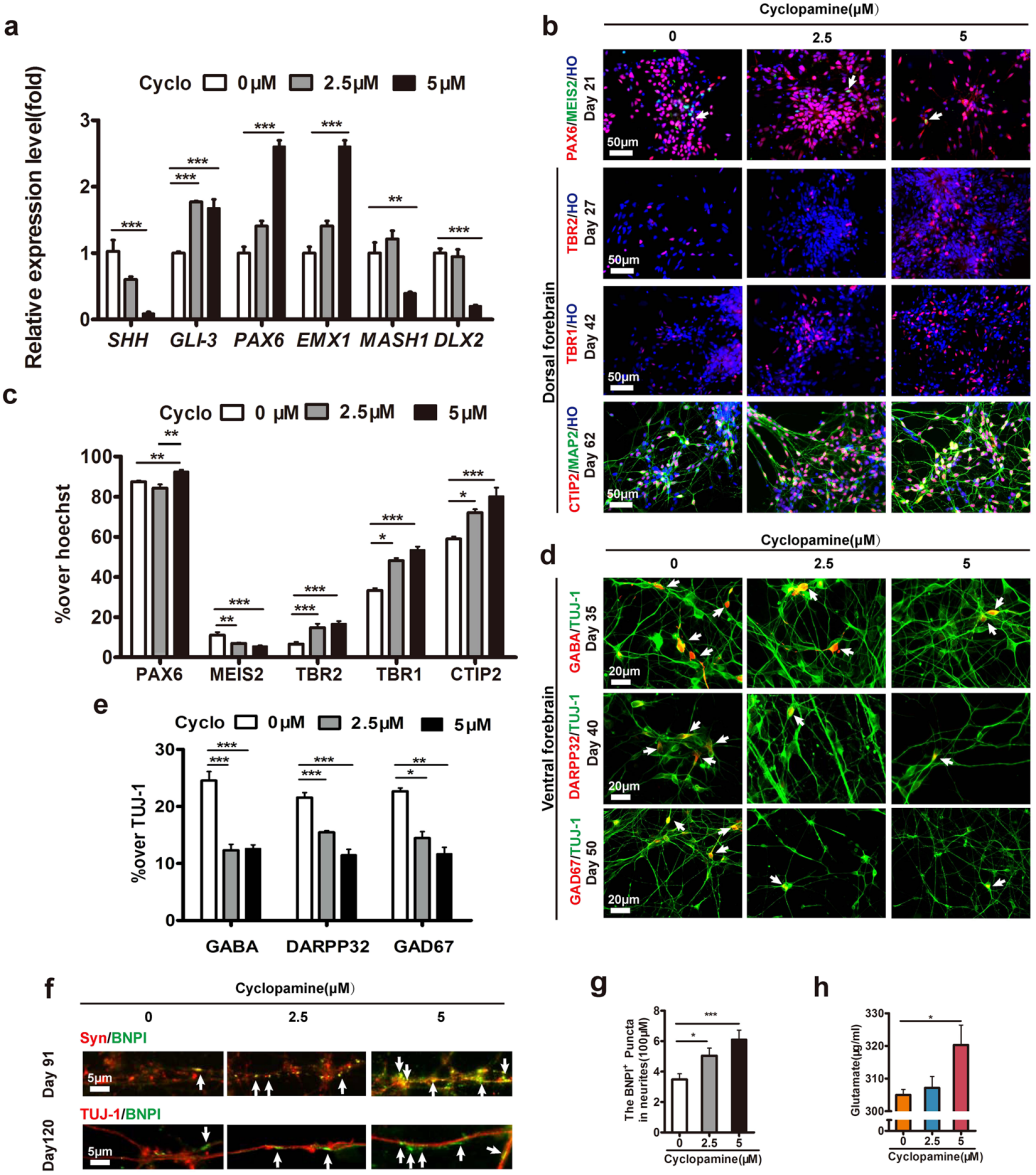
Specification of enriched glutamatergic neurons from hESCs under the high concentration of cyclopamine. (**a**) Fold change of the specific mRNA levels of the *SHH*, *GLI-3*, dorsal forebrain (*PAX6*, *EMX1*) and ventral forebrain (*MASH1*, *DLX2*) at d17. **p < 0.01; ***p < 0.001. n = 3 in each condition. (**b**) Expression of the regionalization markers of the dorsal forebrain (PAX6, TBR2, TBR1, CTIP2), ventral forebrain (MEIS2) from d21 to d62. The arrows indicate the LGE cells co-expressed PAX6 with MEIS2. Scale bar, 50 μm. (**c**) Percentage of the total cells expressing neuronal regionalization markers from d21 of differentiation. *p < 0.05; **p < 0.01; ***p < 0.001. More than 1,500 cells from random fields were manually counted in each condition. The data are presented as the mean ± s.e.m, n ≥ 3 in each condition. (**d**) Expression of the GABAergic neurons related markers GABA (arrows), DARPP32 (arrows), GAD67 (arrows) from d35. Scale bar, 20 μm. (**e**) Quantification of the GABAergic neurons related markers GABA, DARPP32, and GAD67 in total number of neurons. *p < 0.05; **p < 0.01; ***p < 0.001. More than 500 neurons from random fields were manually counted in each condition. The data are presented as the mean ± s.e.m, n ≥ 3 in each condition. (**f**) After d91, BNPI^+^ (arrows) puncta were clearly observed in TUJ-1^+^ neurites and co-localized with synaptophysin^+^ puncta. Scale bar, 5 μm. (**g**) The density of BNPI^+^ puncta in TUJ-1^+^ neurites. *p < 0.05, ***p < 0.001. More than 38,800 μm of TUJ-1^+^ neurites from random fields were manually counted in each condition. The data are presented as the mean ± s.e.m, n = 3 in each condition. (**h**) Glutamate Assay Kit was performed to detect the concentration of glutamate after d180 of differentiation. *p < 0.05. The data are presented as the mean ± s.e.m, n = 3 in each condition.

Because GABA^+^ neurons originated from ventral telencephalon (Supplementary Fig. S3a)^33–36^, the inhibition of endogenous SHH might be a strategy to reduce GABA^+^ neurons. To test whether the antagonist of SHH cyclopamine inhibited human ventral patterning, we firstly examined the dosage of cyclopamine in hESC-derived neural epithelium^23, 37^. 0 μM, 2.5 μM or 5 μM cyclopamine was respectively applied to hESC-derived neural epithelium cultures from day 10 to day 25 (Supplementary Fig. S3b). The *SHH* mRNA levels were decreased in response to cyclopamine in a dose-dependent manner at day 17 (Fig. 1a). The expression of the *SHH* repressor *GLI-3* and the dorsal cortical markers *PAX6* and *EMX1* were increased in the present of cyclopamine. Furthermore, the expression of ventral transcriptional factors *MASH1* and *DLX2* were decreased with the treatment of 5 μM cyclopamine (Fig. 1a). These results suggested that ventral induction of hESC-derived progenitors were inhibited by high concentration of cyclopamine. Consistently, the number of PAX6-positive cells that examined by immunostaining was increased by cyclopamine treatment in a dose-dependent manner (Fig. 1b,c). In contrast, the population of MEIS2^+^ cells decreased (Fig. 1b,c), although the FOXG1^+^ and TUJ-1^+^ cells were not altered (Supplementary Fig. S3c,e). Thus, cyclopamine effectively inhibited ventral telencephalic differentiation.

### Cyclopamine enhanced the generation of glutamatergic neurons effectively

Next, we determined the cortical transcription factors TBR2 and TBR1 were elevated in the presence of 5 μM cyclopamine (Fig. 1b,c). The percentage of CTIP2 was increased to 80.26 ± 4.33% (Fig. 1b,c). In contrast, the number of GABAergic neurons decreased to 12.60 ± 0.66% with cyclopamine treatment (Fig. 1d,e), and partially expressed MEIS2 (Supplementary Fig. S3c). Consistently, DARPP32 and GAD67 were also significantly reduced (Fig. 1d,e). The results suggested that cyclopamine promoted the population of dorsal cell by inhibiting ventral differentiation.

To test whether hPSC-derived glutamatergic neurons became maturation, we examined the expression of MAP2, synaptophysin (Syn) and BNPI (vGLUT). The BNPI^+^ puncta structure was barely observed in the culture before day 80 (data not shown). After 90 days, the glutamatergic neuronal marker BNPI^+^ puncta was co-labeled with MAP2 (Supplementary Fig. S3d), synaptophysin, or TUJ-1 respectively (Fig. 1f). The BNPI^+^ puncta density was increased in a cyclopamine dose-dependent manner in TUJ-1^+^ neurites and synaptophysin^+^ puncta (Fig. 1f,g). Furthermore, cyclopamine treated cells showed a higher percentage of SATB2 (42.47 ± 2.63%) in comparison with untreated group(28.12 ± 2.93%) (Supplementary Fig. S3d,f). To evaluate the function of cyclopamine treated hPSC-derived cortical glutamatergic neurons, we examined the synthesized and released glutamate from hESC-derived glutamatergic neurons after 180 days of differentiation. The glutamate release was significantly increased in 5 μM cyclopamine-treated glutamatergic neurons (Fig. 1h). Moreover, the expression of vGLUT protein was elevated in the 5 μM cyclopamine group by western blotting at day 180 (Supplementary Fig. S3g). Taken together, these results showed that matured and functional glutamatergic neurons were generated from hESCs in the presence of 5 μM cyclopamine.

### The application of cyclopamine at early time point significantly enriched the population of glutamatergic neurons

**Figure 2:**
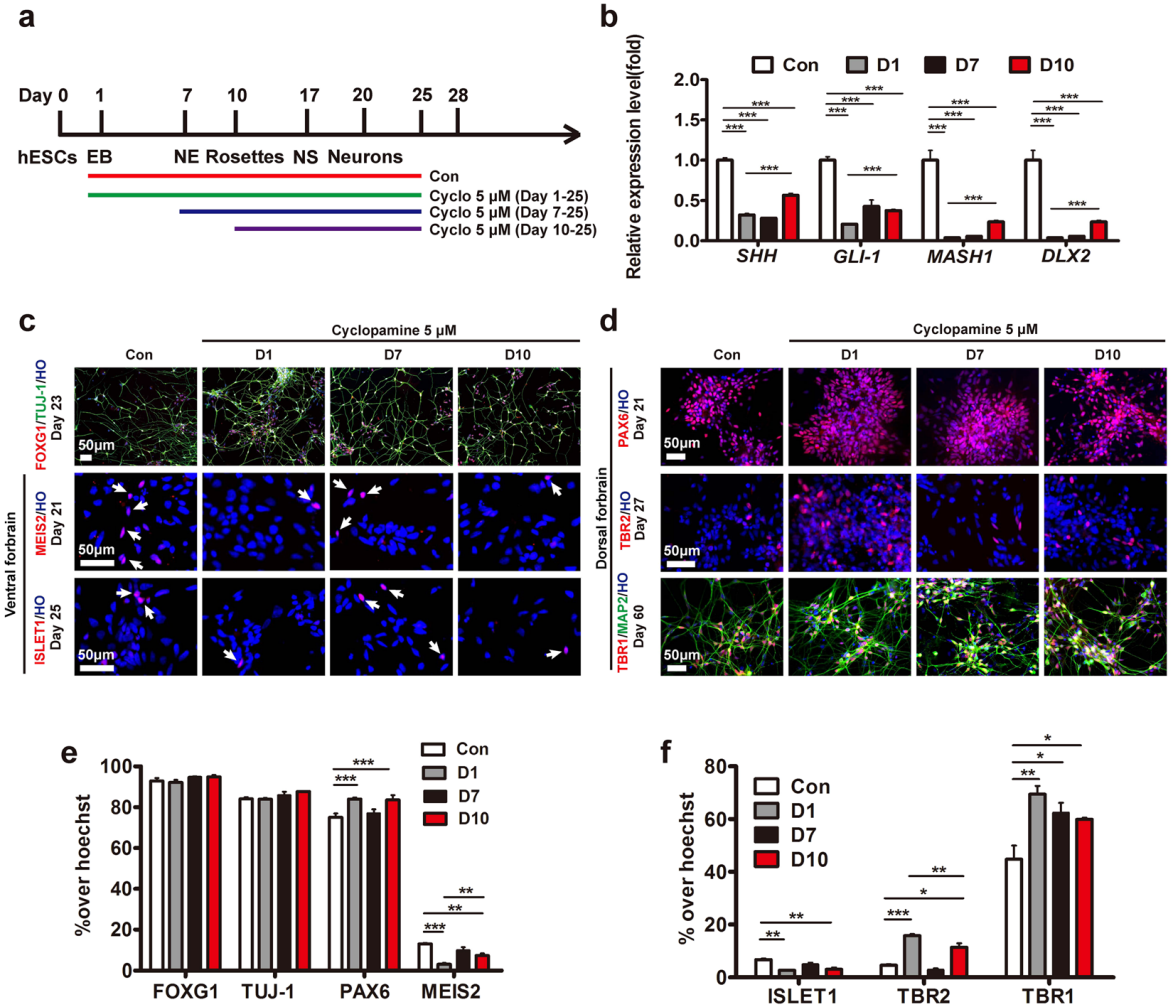
Early application of cyclopamine inhibited the generation of ventral neurons. (**a**) Timeline of differentiating hESCs to forebrain cortical neurons under the different time windows of cyclopamine. (**b**) Fold change of the specific mRNA level of the *SHH*, *GLI-1*, and ventral forebrain (*MASH1*, *DLX2*) at d17. ***p < 0.001. The data are presented as the mean ± s.e.m, n = 3 in each condition. (**c**) Expression of the regionalization markers of the forebrain (FOXG1), and ventral forebrain (MEIS2, ISLET1) (arrows) among the neuron (TUJ-1) from d21 to d25. Scale bar, 50 μm. (**d**) Expression of the cortical neuron markers including PAX6, TBR2, TBR1 from d21 to d60. Scale bar, 50 μm. (**e**) and (**f**) Proportion of the total cells expressing neuronal regionalization markers from d21. *p < 0.05; **p < 0.01; ***p < 0.001. More than 1,500 cells from random fields were manually counted in each condition. The data are presented as the mean ± s.e.m, n ≥ 3 in each condition.

**Figure 3:**
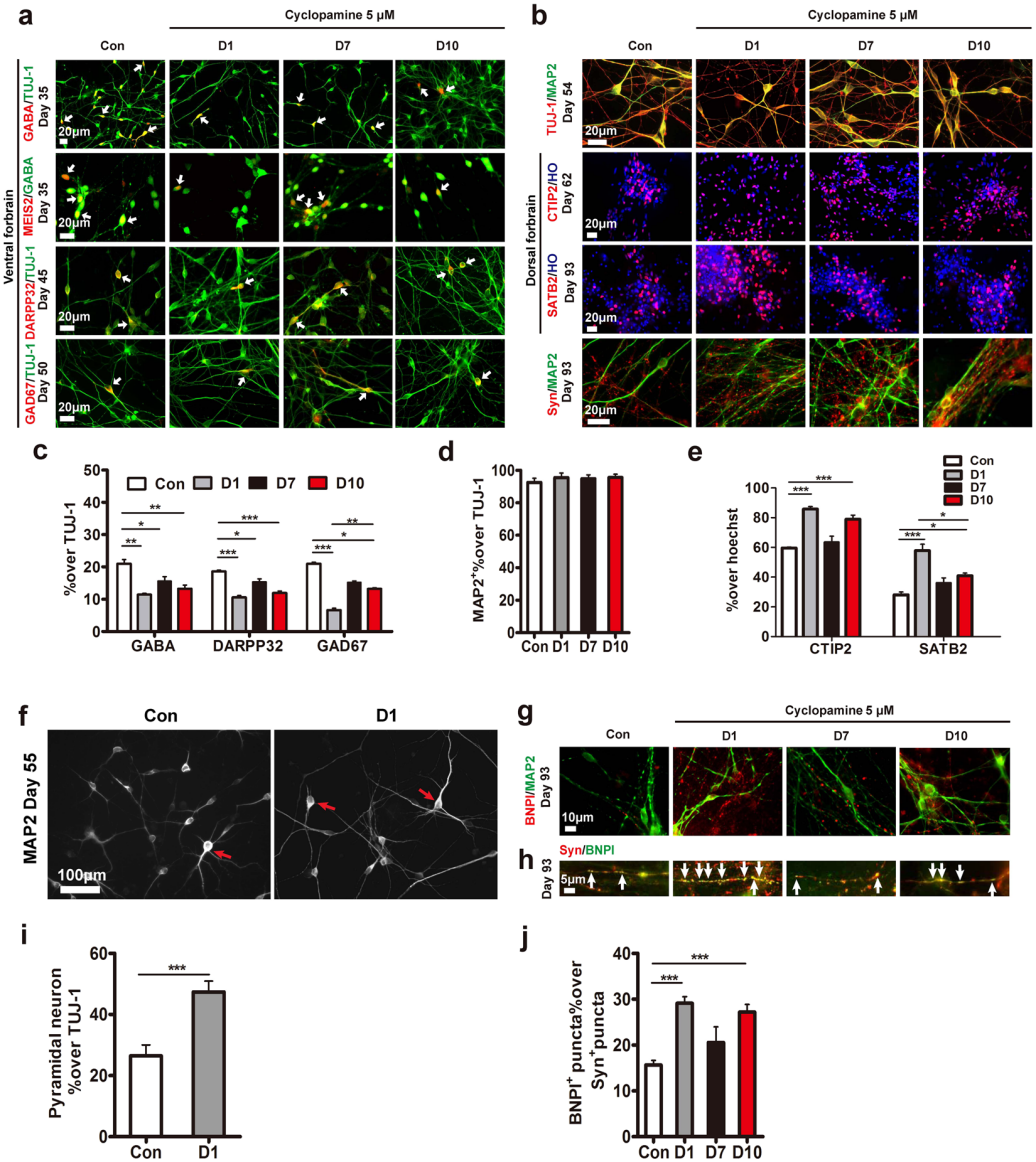
Early application of cyclopamine enriched the generation of glutamatergic neurons. (**a**) Expression of the GABAergic neurons related markers GABA (arrows), DARPP32 (arrows), and GAD67 (arrows) and LGE marker MEIS2 (arrows) from d35. Scale bar, 20 μm. (**b**) Expression of specific markers of mature neurons (MAP2), cortical neurons (CTIP2 and SATB2) and presynaptic protein synaptophysin from d54. Scale bar, 20 μm. (**c**) Proportion of GABAergic neurons related markers, including GABA, DARPP32, and GAD67, from d35. *p < 0.05; **p < 0.01; ***p < 0.001. More than 500 neurons from random fields were manually counted in each condition. The data are presented as the mean ± s.e.m, n ≥ 3 in each condition. (**d**) Proportion of MAP2^+^ neurons showed no significant differences in all groups. More than 500 neurons from random fields were manually counted in each condition. The data are presented as the mean ± s.e.m, n = 3 in each condition. (**e**) Proportion of CTIP2^+^ and SATB2^+^ cortical neurons in total cells. *p < 0.05; ***p < 0.001. More than 1,500 cells from random fields were manually counted in each condition. The data are presented as the mean ± s.e.m, n ≥ 3 in each condition. (**f**) Distinctive pyramidal morphology (arrows) was observed for MAP2^+^ mature neurons at d55. Scale bar, 100 μm. (**g**) At d93, mature cortical neurons expressed vesicular glutamate transporters BNPI^+^ puncta. Scale bar,10 μm. (**h**) At d93, BNPI^+^ puncta (arrows) were clearly observed and co-localized with synaptophysin^+^ puncta. Scale bar, 5 μm. (**i**) Significantly higher cyclopamine treatment neurons displayed pyramidal morphology. ***p < 0.001. More than 500 neurons from random fields were manually counted in each condition. The data are presented as the mean ± s.e.m, n = 3 in each condition. (**j**) Proportion of BNPI^+^ puncta in synaptophysin^+^ puncta. ***p < 0.001. More than 9,000 synaptophysin^+^ puncta from random fields were manually counted in each condition. The data are presented as the mean ± s.e.m, n = 3 in each condition.

We next optimized the time window of cyclopamine treatment. hPSCs were treated with 5 μM cyclopamine starting from day 1, day 7 or day 10 (Fig. 2a). hPSCs treated from day 1 showed the most effective inhibition of *SHH*, *GLI-1* (downstream effectors of SHH pathway), *MASH1* and *DLX2* (Fig. 2b). The numbers of FOXG1^+^/TUJ-1^+^ cell were similar among those groups, which suggested that cyclopamine did not alter telencephalic neuronal patterning (Fig. 2c,e). hPSCs treated from day 1 showed an increased proportion of the PAX6^+^ cells but reductions of MEIS2^+^ (3.03 ± 0.61%) and ISLET1^+^ (2.68 ± 0.23%) cells (Fig. 2c–f). Furthermore, the expression of TBR2 cells showed a significantly increased (15.75 ± 0.58%) in the day-1 group, displaying more effective differentiation of cortical progenitors (Fig. 2d,f). Continuous differentiation of neurons over a month, 69.46 ± 3.06% of TUJ-1^+^ cells expressed TBR1 in the day-1 group, while the control group generated only 44.78 ± 5.20% TBR1^+^ neurons (Fig. 2d,f). The ventral GABA neurons decreased to 11.38 ± 0.47% in the day-1 group, whereas there were 20.99 ± 1.25% GABA neurons in the control groups. In addition, there was a significant decrease in the number of DARPP32^+^ cells and GAD67^+^ cells in the day-1 group compared to the other groups (Fig. 3a,c), suggesting the inhibition of GABA neurons with early treatment of cyclopamine was efficient.

With maturation, more than 90% of TUJ-1^+^ cells were positive for MAP2 in each group (Fig. 3b,d). After day 55, more than 48% MAP2^+^ neurons in the day-1 group exhibited pyramidal neuronal morphology, with an apical dendrite and multiple basal dendrites, compared to the control (26.50 ± 3.59%) (Fig. 3f,i)^22, 38^. Following over 90-day differentiation, significant more neurons expressed BNPI^+^ puncta, which co-localized with synaptophysin^+^ puncta in the day-1 group (Fig. 3g,h,j). Moreover, we found dramatically more CTIP2^+^ (85.79 ± 1.68%) cortical neurons in the day-1 group compared to the control group (59.54 ± 0.69%) (Fig. 3b,e). In addition, the expression of SATB2 was increased to 57.81 ± 4.30% with cyclopamine treatment, compared to 28.04 ± 1.94% in the control group (Fig. 3b,e). In addition, we observed the similar levels of proliferative markers of KI67 and nucleotide analogs (ethynyl deoxyuridine, EdU), which was added for 10 hours, in the control and day-1 group respectively (Supplementary Fig. S3h,i). The results revealed the cyclopamine enhanced glutamatergic neurons by suppression of ventralization rather than inhibiting cell division. Taken together, these results revealed that early treatment of cyclopamine promoted the generation of glutamatergic neurons and decreased the GABAergic neurons.

### Cyclopamine-enriched Down syndrome patient iPSC-derived glutamatergic neurons exhibited the reduction of vesicular glutamate transporters

**Figure 4:**
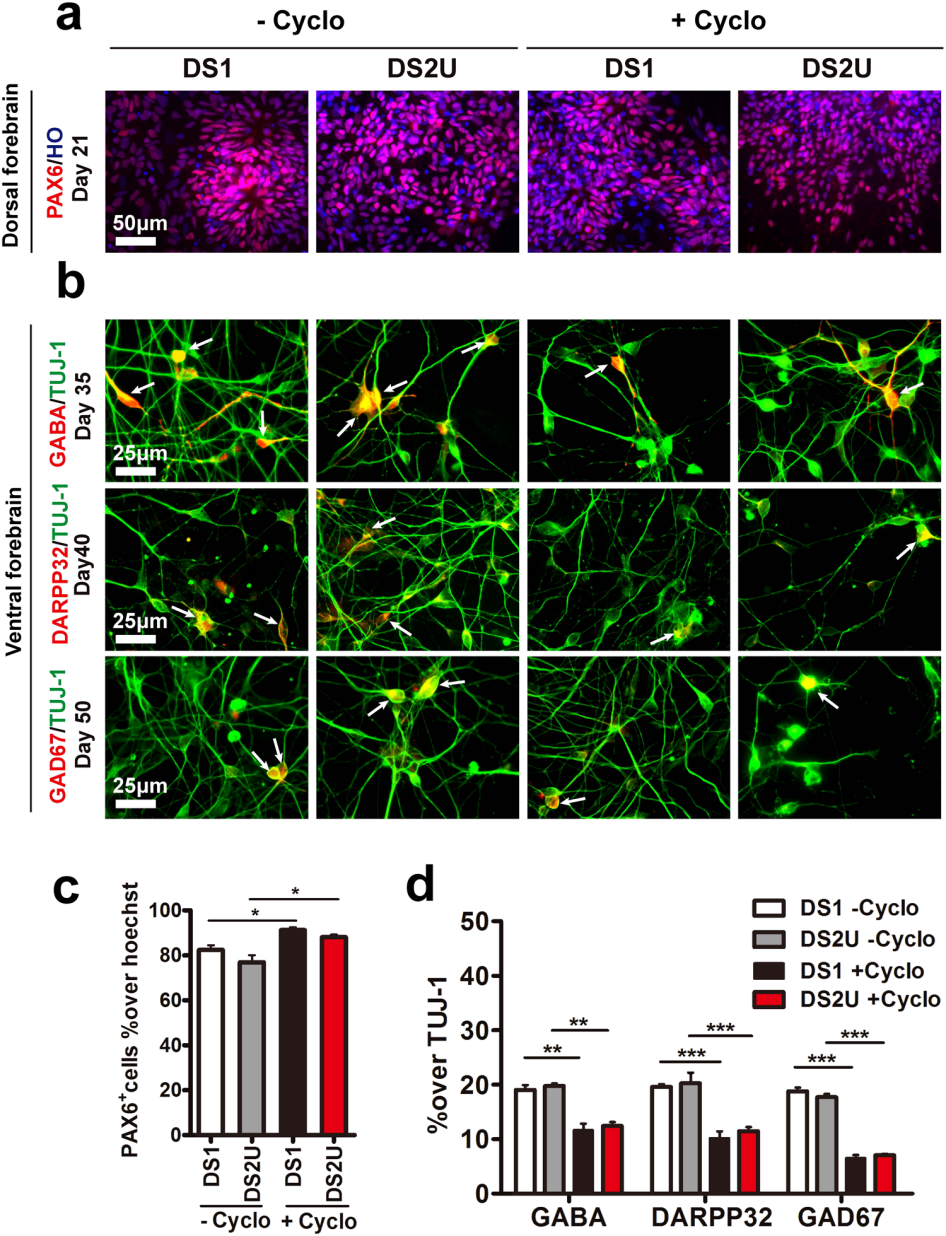
Application of cyclopamine inhibited the ventral patterning from Down Syndrome patient-iPSCs. (**a**) Expression of marker for dorsal forebrain (PAX6) from d21. Scale bar, 50 μm. (**b**) Expression of markers for ventral forebrain neurons (GABA, DARPP32, GAD67) (arrows) from d35. Scale bar, 25 μm. (**c**) Proportion of PAX6^+^ cells in total cells. *p < 0.05. More than 1,500 cells from random fields were manually counted in each condition. The data are presented as the mean ± s.e.m, n = 3 in each condition. (**d**) Significantly fewer GABAergic related neurons in cyclopamine treatment groups. **p < 0.01; ***p < 0.001. More than 500 neurons from random fields were manually counted in each condition. The data are presented as the mean ± s.e.m, n ≥ 3 in each condition.

Down syndrome (DS) patients have severe intellectual disability because of trisomic chromosome 21(Ts21)^39, 40^. Previous studies showed decreased cortical areas in DS patients^41, 42^. Abnormal development of glutamatergic neurons may connect to intellectual disability. To gain insight into cortical glutamatergic neurons in DS, we applied this optimized protocol to differentiate cortical glutamatergic neurons from DS-patients iPSCs lines DS1 and DS2U^19^. All iPSCs lines were positive for alkaline phosphatase (AP) (Supplementary Fig. S4a), and expressed the pluripotent factors OCT4 and SOX2 (Supplementary Fig. S4b). Karyotype analysis showed that the chromosome 21 of DS1 iPSCs is trisomic, whereas DS2U is disomic for human chromosome 21 (Supplementary Fig. S4c). By following our optimized protocol, iPSCs differentiated to neuroepithelium as demonstrated by increased *PAX6* expression after d10, which was exhibited higher levels in cyclopamine treatment groups compared the control groups (Supplementary Fig. S4d). The mRNA level of pluripotent gene *NANOG* was down-regulated during neural differentiation, and the expression levels of mesodermal marker *BRACHYUR*Y and endodermal marker *SOX17* were limited when the cells were differentiated to NE (Supplementary Fig. S4d). With the treatment of 5 μM cyclopamine from day 1 to day 25, both iPSCs lines generated significant more PAX6^+^ cells (90%) that examined by immunostaining at day 21 (Fig. 4a,c), but decreased GABA^+^, DARPP32^+^ and GAD67^+^ neurons (~10%) after day 35 (Fig. 4b,d), which was consistent with the results from H9 hESCs.

**Figure 5:**
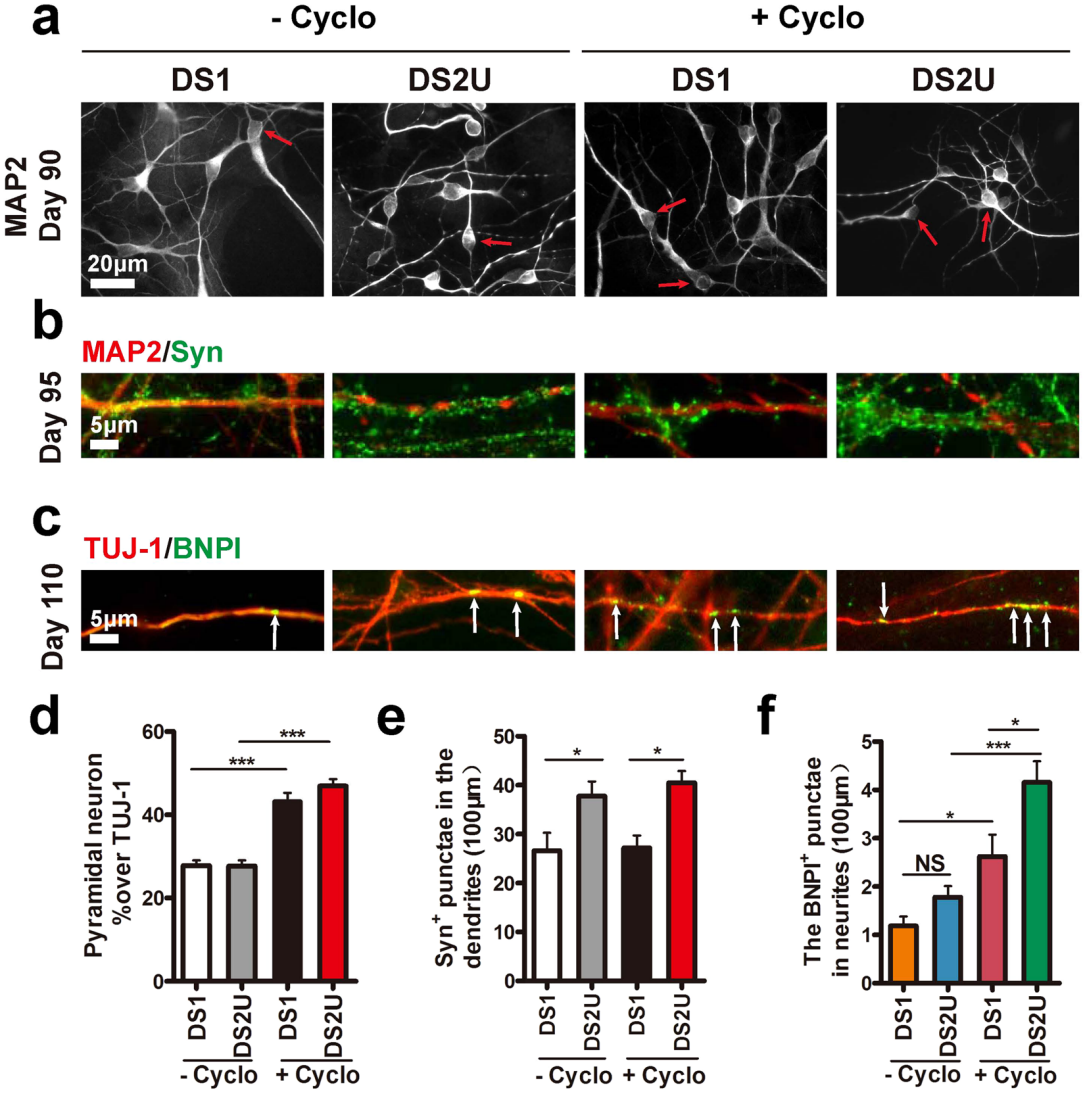
Cyclopamine enriched Down syndrome patient iPSC-derived glutamatergic neurons that exhibited the reduction of vesicular glutamate transporters. (**a**) Mature cortical neurons exhibited pyramidal morphology (arrows) from d90. Scale bar, 20 μm. (**b**) Mature neurons expressed MAP2 and synaptophysin from d95. Scale bar, 5 μm. (**c**) Glutamatergic neurons expressed BNPI (arrows) from d110. Scale bar, 5 μm. (**d**) Proportion of pyramidal neurons in total neurons. ***p < 0.001. More than 500 neurons from random fields were manually counted in each condition. The data are presented as the mean ± s.e.m, n = 3 in each condition. (**e**) Fewer synaptophysin^+^ puncta in DS1 neurons compared to DS2U neurons. *p < 0.05. More than 26,000 μm of MAP2^+^ dendrites from random fields were manually counted in each condition. The data are presented as the mean ± s.e.m, n = 3 in each condition. (**f**) Less BNPI^+^ puncta in DS1 compared to DS2U cortical neurons with cyclopamine treatment. *p < 0.05; ***p < 0.001. More than 16,000 μm of TUJ-1^+^ neurites from random fields were manually counted in each condition. The data are presented as the mean ± s.e.m, n = 3 in each condition.

In the cyclopamine-treatment groups, both DS1 and DS2U iPSC-derived cortical glutamatergic neurons showed that more than 43% neurons displayed pyramidal morphology compared to approximately 27% pyramidal morphology in the untreated groups (Fig. 5a,d). These results indicated that cyclopamine effectively promoted the generation of dorsal glutamatergic neurons by inhibiting ventral GABAergic neurons in both iPSCs lines.

Next, we tested the number of pre-synaptic puncta in those iPSC-derived DS neurons. A significant reduction in synaptophysin^+^ density between DS1 and DS2U neurons was observed in both cyclopamine-treated and -untreated groups (Fig. 5b,e). We showed that BNPI^+^ puncta was significant increased by cyclopamine treatment in both iPSCs lines (Fig. 5c,f). More importantly, we found a significant reduction in BNPI^+^ density in trisomy iPSC-derived cortical neurons with the treatment of cyclopamine, compared to the euploid control, which were not observed in the untreated groups (Fig. 5c,f).

Taken together, these results suggested that cyclopamine promoted the generation of glutamatergic neurons from DS iPSCs and reflected the vesicular glutamate transporter defects of glutamatergic neurons in Down syndrome. This new strategy may facilitate to generate cortical glutamatergic neurons from the patient iPSCs for disease modeling.

## Discussion

Cortical glutamatergic neurons regulated many critical brain functions. The availability of human glutamatergic neurons *in vitro* would provide a useful platform to study the human cortical development, neurological disease, and cell therapy. Recently, cortical glutamatergic neurons were successfully generated from hPSCs, including one study presented a temporal gene expression data set encompassing cerebral cortical development from hESCs by using RNA-Seq and global temporal transcriptome^43^. However, a couple of key issues prevented the application of hPSC-derived cortical glutamatergic neurons to clinical translation. Firstly, a number of GABAergic neurons (20~40%) were mixed in the hPSC-derived glutamatergic neurons^17–19^. Secondly, heterogeneity of hPSC-derived neurons was observed in the culture system because of different stage of differentiation. Last but not the least, hPSC-derived neurons were largely immature neurons. To enhance the purity of hPSC-derived glutamatergic neurons, we applied the SHH inhibitor cyclopamine to inhibit the differentiation of ventral cells. In this study, we compared multiple concentrations of cyclopamine, and we showed that 5 μM cyclopamine was the optimal condition for inhibiting the ventral cells. We also compared the multiple time points for SHH inhibitor treatment and showed that SHH inhibitor treatment from day 1 to day 25 promoted dorsal patterning significantly.

Ventral inhibition with the application of cyclopamine in the derivation of glutamatergic neurons from mouse embryonic stem cells was efficient^22, 23, 44^. However, the significant ventral inhibition with cyclopamine treatment was not successful in hPSCs^24–26^. Many efforts have been put to test the SHH inhibitor on the differentiation of glutamatergic neurons from hPSCs. The application of 1 μM cyclopamine from day 17 to day 24^24^, from day 10 to day 28^25^ or from day 2 to day 10 during differentiation^26^ was tested. But the percentage of hPSCs-derived glutamatergic neurons was not affected. One study used the combination of cyclopamine and morphogen FGF-2 for increasing the dorsal patterning and modeled Alzheimer’s disease^45^. In our study, we comprehensively compared the treatment of cyclopamine at different dosage and time window, and analyzed the expression of dorsal and ventral marker under the different treatment of cyclopamine. We showed that application of 5 μM cyclopamine from day 1 to day 25 promoted dorsal patterning significantly. The effect of cyclopamine on cortical and ventral developmental transcriptional factors during differentiation was also carefully investigated and cyclopamine optimized hPSCs-derived the glutamatergic neurons showed the cortical characteristic. Furthermore, by using our current method, the GABAergic neurons were significantly reduced to 12% upon anti-GABA immunostaining. In addition, we validated the reduction of ventral GABAergic neurons by testing additional GABAergic markers (GAD67 and DARPP32) and ventral transcriptional factors (*SHH*, *GLI-1*, *MASH1* and *DLX2* and MEIS2 and ISLET1).

An efficient differentiation system would allow the iPSCs model “bona-fide” disease symptom, which occurred *in vivo* ^46, 47^. However, very few studies showed the deficits in trisomy iPSC-derived glutamatergic neurons. One key issue is that the purity of hPSCs-derived glutamatergic neurons were low and a number of non-glutamatergic neurons were mixed in the system. The application of SHH inhibitor provided a system to generate higher percentage of glutamatergic neurons from hPSCs. With the increased purity of hPSCs-derived glutamatergic neurons, trisomy iPSC-derived neurons exhibited decreased BNPI^+^ puncta in comparison with the euploid control, which suggested the developmental deficits of glutamatergic neurons in DS cortex. These significant phenotypes were not observed in trisomy iPSC-derived neurons without the enriched hPSCs-derived glutamatergic neurons by applying cyclopamine.

In conclusion, we significantly enhanced the percentage of hPSCs-derived glutamatergic neurons by only applying one SHH inhibitor cyclopamine into hPSC system. More excitingly, by using this method, we showed that the trisomy iPSC-derived neurons exhibited the decrease of BNPI^+^ puncta. With the increased purity of glutamatergic neurons from hPSCs, our current paradigm may provide an optimal platform for glutamatergic neuron-associated studies, such as human cortical development, neurological disease studies, and cell-based transplantation therapy.

## Materials and Methods

### hPSCs maintenance and passage

All the studies were approved by Research Ethic Committee in Nanjing Medical University (NO. (2006) 326). Human ESCs (H9, passages 60–80, WiCell Agreement No. 16-W0060) and human iPSCs derived from Down syndrome patient (DS1 passages 50–60 and DS2U passages 60–69, human iPSCs were kind gifts of Dr. Anita Bhattacharyya, Waisman Center and WiCell Research Institute^19^) were maintained under a feeder-free condition with Essential 8 medium (Gibco) on vitronectin (Life Technologies)-coated plates as previously described in the standard protocol http://www.wicell.org. Colonies were passaged using EDTA (Lonza) when they became 80% confluent.

### Differentiation of cortical glutamatergic neurons from hPSCs

To initiate differentiation, hPSCs colonies were treated with 1 Uml^−1^ dispase (Life Technologies) for 2–5 minutes in an incubator as previously described in detail^30^. After detachment, the cells were suspended in neural induction medium (NIM). The colonies were aggregated to embryonic bodies (EBs) for seven days. TGF-β inhibitors SB431542 (Tocris Bioscience, 2 μM) and BMP inhibitor DMH-1 (Tocris Bioscience, 2 μM) could be added from day 1 to day 6 to enhance neural induction. From day 7 to day 16, the EBs were attached onto six-well plates for rosette formation. The rosette structure was observed since day 10. On day 16, the rosettes were detached by a pipette tip and resuspended in NIM containing B27 (Gibco), which may reduce the damage that caused by the mechanical pipette. On the next day, the cells formed neural spheres (NS). For neuronal differentiation, NS were dissociated with TrypLE (Life Technologies) and attached onto a coverslip that precoated with poly-L-ornithine (Sigma) and matrigel (BD Biosciences) at a density of 40,000 cells per coverslip in 24-well plates. After 2 hours, 500 μl NIM was added into each well, including B27, 1 μM cAMP (Sigma), and 10 nml^−1^ BDNF (PeproTech) on day 20. The medium was refreshed every 5–6 days. To optimize the differentiation system, 0–5 μM cyclopamine (Merck) was added to the culture from day 1, 7, and 10 to day 25 as illustrated in Figs 2a and S3b.

### Immunocytochemistry

Neurons were fixed with cold 4% paraformaldehyde (PFA) for 30 minutes. After being washed with PBS 3 times, the neurons were treated with 0.2% Triton X-100 for 10 minutes and incubated in 10% donkey serum for 1 hour at room temperature. The cells were incubated in primary antibodies (Supplementary Table S1), which were prepared in 5% donkey serum and 0.1% Triton X-100, overnight at 4 °C. On the next day, the cells were washed with PBS for 3 times at 10-minute intervals. Secondary antibodies were prepared in 5% donkey serum and the cells were incubated for 30 minutes at room temperature. The nuclei were counterstained with Hoechst 33258 dye (Life Technologies). Next, the cells were washed with PBS 3 times and mounted for fluorescent imaging.

### Quantitative real-time polymerase chain reaction (qPCR)

Cells were collected on day 17 and dissociated with TRIzol reagent. The RNA was extracted from neurospheres and diluted to 1 μg with DAPC-treated water. SuperScript III First-Strand system (Life Technologies) was used to synthesize the cDNA. qPCR was performed in a 20-μl reaction system, which included 4 μl cDNA, 2 μl random primers (Supplementary Table S2), 2 μl ddH_2_O and 10 μl 2X SYBR Green RCR Master Mix (Roche). Glyceraldehyde-3-phosphate dehydrogenase (GAPDH) was used as a housekeeping gene.

### Western blotting analyses

Protein samples were lysed in RIPA lysis buffer (Beyotime Institute of Biotechnology) with protease inhibitor cocktail and the protein concentration was measured using the BCA kit (Shanghai Yeasen Biotechnology). The Western blot was performed as previously described using vGLUT (1:1000, Synaptic System) and GAPDH (1:4000, Kangchen) as primary antibodies.

### Glutamate concentration measurement

Approximately 1 ml of collected day-180-neuron medium was added to 5 ml reagent. The samples were centrifuged at 8000 × *g* for 10 minutes to remove insoluble material. Next, 250 μl supernatant was aspirated into a 1.5-ml Eppendorf tube and added to 50 μl of reagent 2. The mixture was then transferred into the 90 °C water bath for 20 minutes. After running under cool water, the sample was added to 96-well plates (100 μl/well). The glutamate concentration was determined using a microplate reader (Molecular Devices SpectraMax M2). Reagents 1 and 2 were obtained from Qiyi Biological Technology.

### Synaptic and BNPI Density

We followed previous reported method^19^ to detect synaptic and BNPI density. Over-90-day-old hPSCs-derived neurons were labeled with anti-synaptophysin (Syn), anti-vesicular glutamate transporters (BNPI), anti-MAP2 or anti-TUJ-1 (Supplementary Table S1). For pre-synaptic density analysis, we randomly traced MAP2^+^ dendrites manually and converted to synaptophysin images for counting. For BNPI density analysis, random TUJ-1^+^ neurites were manually traced and the field was transferred to BNPI images for counting. For BNPI/Synaptophysin analysis, puncta for each image were counted separately in one region and then merged to determine co-localization percentage.

### Morphology of pyramidal neurons

A pyramidal morphology index (PIM) was used to objectively measure morphology of pyramidal neurons as previous reported method described^22^. According to the previous reported method, the ratio between the width of the largest dendrite (LD) divided by the number of neurites (N) was determined as PIM. If the value of PIM is greater than 1.2, the neuron was considered as pyramidal neurons.

### Statistical analysis

To quantify the results, a fluorescence microscope (Eclipse 80i) was used to capture the images. At least 12 random fields were chosen, and more than 5,000 cells of each cell line were quantified and more than 3 independent differentiation assays were performed. The images were constructed using Adobe Illustrator CS5. Values were obtained using paired t-test with Image-J software. The data are presented as s.e.m, and p < 0.05 was considered significant.

## Electronic supplementary material

Supplementary information
