# Supplementary material for: Enhanced derivation of human pluripotent stem cell-derived cortical glutamatergic neurons by a small molecule
Source: Sci Rep. 2017 Jun 12;7:3282. doi: 10.1038/s41598-017-03519-w (PMC5468244; doi:10.1038/s41598-017-03519-w)
Supplement: Supplementary file 1 — Supplementary information [file 41598_2017_3519_MOESM1_ESM.pdf]

Supplementary information

Enhanced derivation of human pluripotent stem cell-derived cortical glutamatergic neurons by a small molecule

Shi-Ying Cao[1,2], Yao Hu[1], Cheng Chen[1], Fang Yuan[1], Min Xu[1] , Qi Li[1], Kai-Heng Fang[1], Yaoyu Chen[1], and Yan Liu[1*]

[1]Institute for Stem Cell and Neural Regeneration, School of Pharmacy, Nanjing Medical University, Nanjing, China

[2]Department of Student Affairs, Kangda college of Nanjing Medical University, Lianyungang, China

*Author for correspondence: yanliu@njmu.edu.cn

Supplementary Figure S1

Supplementary Figure S2

Supplementary Figure S3

Supplementary Figure S4

Supplementary Table S1

Supplementary Table S2

Supplementary Figure S1. hPSCs differentiated to neuroepithelial cells

(a) Timeline of differentiating hESCs to forebrain cortical neurons by default differentiation. (b) hPSCs differentiated to neuroepithelial cells, which expressed PAX6, SOX1, SOX2 and KI67, as well as the telencephalic markers FOXG1 and OTX2 at d10. Scale bar, 50 μm.

Supplementary Figure S2. hESC-derived cortical progenitors were mixed with ventral cells by default differentiation

(a) $PAX6^+$ progenitors were mixed with $MEIS2^+$ ventral cells. Scale bar, 50 μm. (b) $TBR2^+$ cells were observed from d27 and Cortical markers TBR1, CTIP2 and SATB2 were detected after d40. Scale bar, 50 μm. (c) Some neurons expressed the ventral marker ISLET1 at d25, and GABAergic neurons were defined by related markers, including GABA, DARPP32, and GAD67 after d35. Scale bar, 50 μm. (d) At d150, neurons expressed the glutamatergic vesicular transporter marker BNPI. Scale bar, 20 μm.

Supplementary Figure S3. High concentration of cyclopamine promoted dorsal patterning

(a) Schematic of a coronal section of the developing fetal forebrain. (b) Timeline of differentiating hESCs to forebrain cortical neurons under different cyclopamine concentrations. (c) Expression of the regionalization markers of the forebrain (FOXG1), neuron (TUJ-1) from d21. Scale bar, 50 μm ; Many $GABA^+$ neurons expressed LGE marker MEIS2 (arrows) from d35. Scale bar, 25 μm (d) At d91, nearly all of the neurons became mature and expressed mature neuronal marker MAP2, pre-synaptic protein marker synaptophysin. Scale bar, 10 μm; At d102, cortical neurons were defined by upper-layer marker SATB2. Scale bar, 100 μm. (e) Percentage of the total cells expressing neuronal regionalization markers from d21 of differentiation. More than 1,500 cells

from random fields were manually counted in each condition. The data are presented as the mean ± s.e.m, n = 3 in each condition. (f) Proportion of $SATB2^+$ neurons in total cells at d102 in culture. *$p < 0.05$. More than 1,500 cells from random fields were manually counted in each condition. The data are presented as the mean ± s.e.m, n = 3 in each condition. (g) Analysis of Western blot product from d180 for control and 5 μM cyclopamine-treated group for vGULT (upper panel) and GAPDH housekeeping gene (lower panel). *$p < 0.05$. The data are presented as the mean ± s.e.m, n = 3 in each condition. (h) At d20, cortical neurons expression of proliferative markers KI67 and EdU, Scale bar, 50 μm. (i) Proportion of $KI67^+$ and EdU^+ neurons in total cells in culture. More than 1,500 cells from random fields were manually counted in each condition. The data are presented as the mean ± s.e.m, n = 3 in each condition.

Supplementary Figure S4. The characteristics of iPSCs

(a) AP staining for iPSCs. Scale bar, 250 μm. (b) iPSCs expressed SOX2 and OCT4. Scale bar, 25 μm. (c) Karyotype analysis for iPSCs.(d) Expression pattern of *NANOG, PAX6, BRACHYURY, SOX17* mRNA during neural differentiation of iPSCs. Data are presented as mean ± s.e.m, n = 3 in each condition.

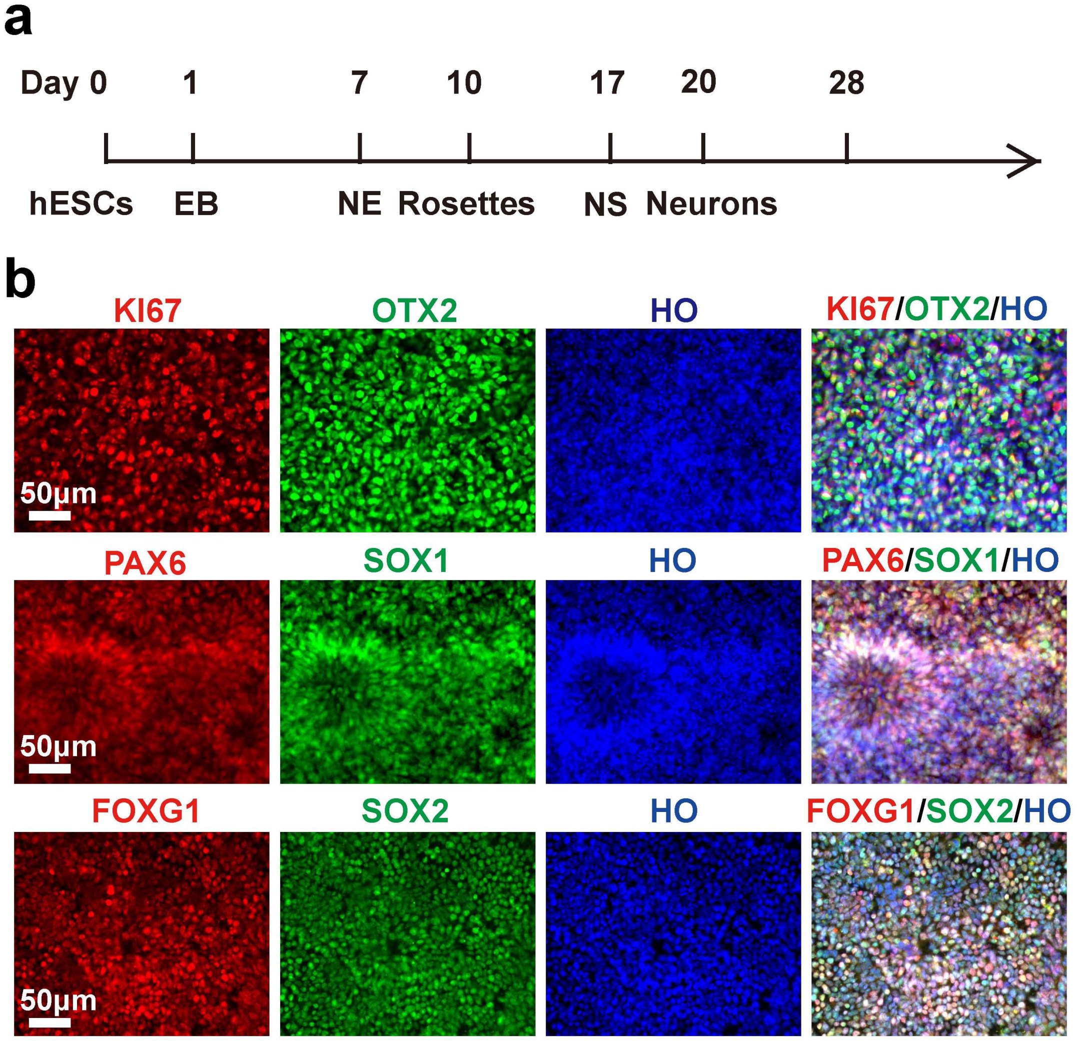
a
Day 0
1
7
10
17
20
28
hESCs
EB
NE
Rosettes
NS
Neurons
b
KI67
OTX2
HO
KI67/OTX2/HO
50μm
PAX6
SOX1
HO
PAX6/SOX1/HO
50μm
FOXG1
SOX2
HO
FOXG1/SOX2/HO
50μm

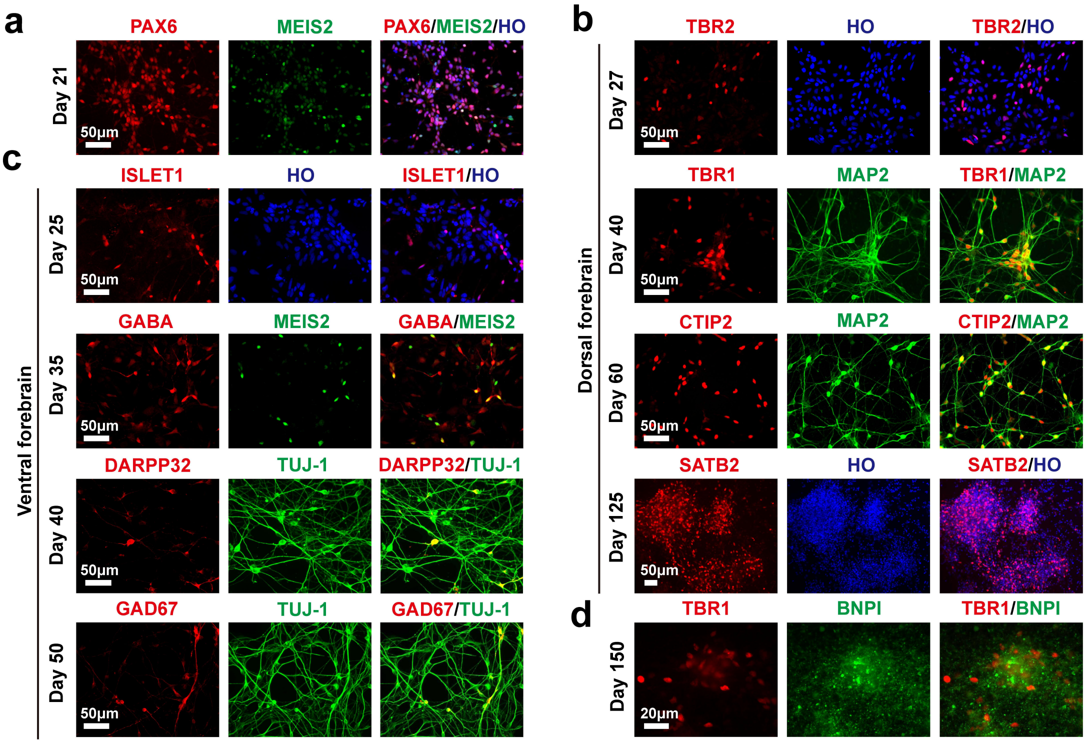

a
PAX6
MEIS2
PAX6/MEIS2/HO
Day 21
50µm
b
TBR2
HO
TBR2/HO
Day 27
50µm
c
ISLET1
HO
ISLET1/HO
Day 25
50µm
TBR1
MAP2
TBR1/MAP2
Day 40
50µm
GABA
MEIS2
GABA/MEIS2
Day 35
50µm
CTIP2
MAP2
CTIP2/MAP2
Day 60
50µm
Ventral forebrain
Dorsal forebrain
DARPP32
TUJ-1
DARPP32/TUJ-1
Day 40
50µm
SATB2
HO
SATB2/HO
Day 125
50µm
GAD67
TUJ-1
GAD67/TUJ-1
Day 50
50µm
d
TBR1
BNPI
TBR1/BNPI
Day 150
20µm

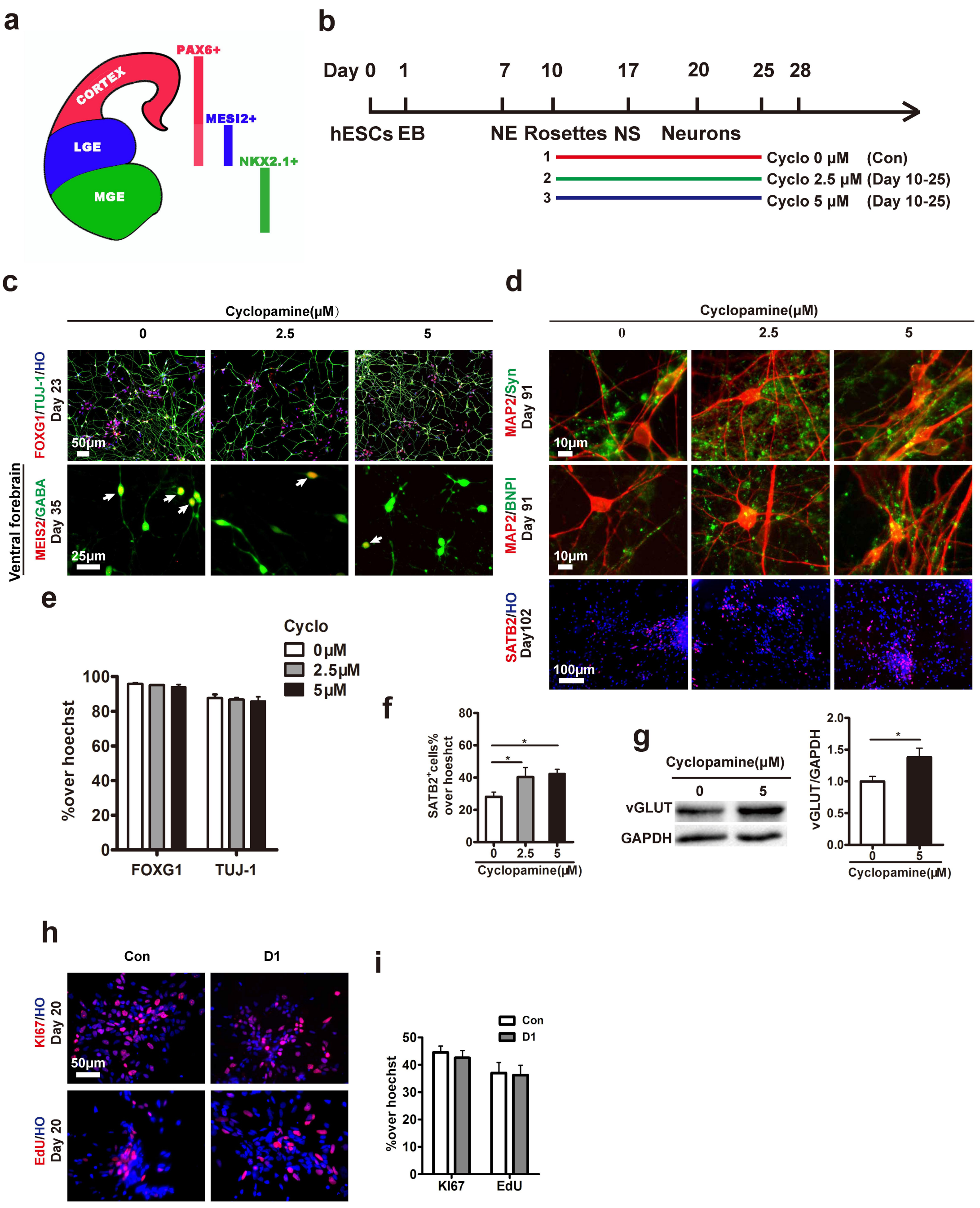
a
CORTEX
LGE
MGE
PAX6+
MESI2+
NKX2.1+
b
Day 0 1 7 10 17 20 25 28
hESCs EB NE Rosettes NS Neurons
1 Cyclo 0 μM (Con)
2 Cyclo 2.5 μM (Day 10-25)
3 Cyclo 5 μM (Day 10-25)
c
Cyclopamine(μM)
0 2.5 5
FOXG1/TUJ-1/HO Day 23
50μm
MEIS2/GABA Day 35
25μm
Ventral forebrain
d
Cyclopamine(μM)
0 2.5 5
MAP2/Syn Day 91
10μm
MAP2/BNPI Day 91
10μm
SATB2/HO Day102
100μm
e
Cyclo
0μM
2.5μM
5μM
%over hoechst
FOXG1 TUJ-1
f
SATB2+cells% over hoeshct
Cyclopamine(μM)
g
Cyclopamine(μM)
0 5
vGLUT
GAPDH
vGLUT/GAPDH
Cyclopamine(μM)
h
Con D1
KI67/HO Day 20
50μm
EdU/HO Day 20
i
Con
D1
%over hoechst
KI67 EdU

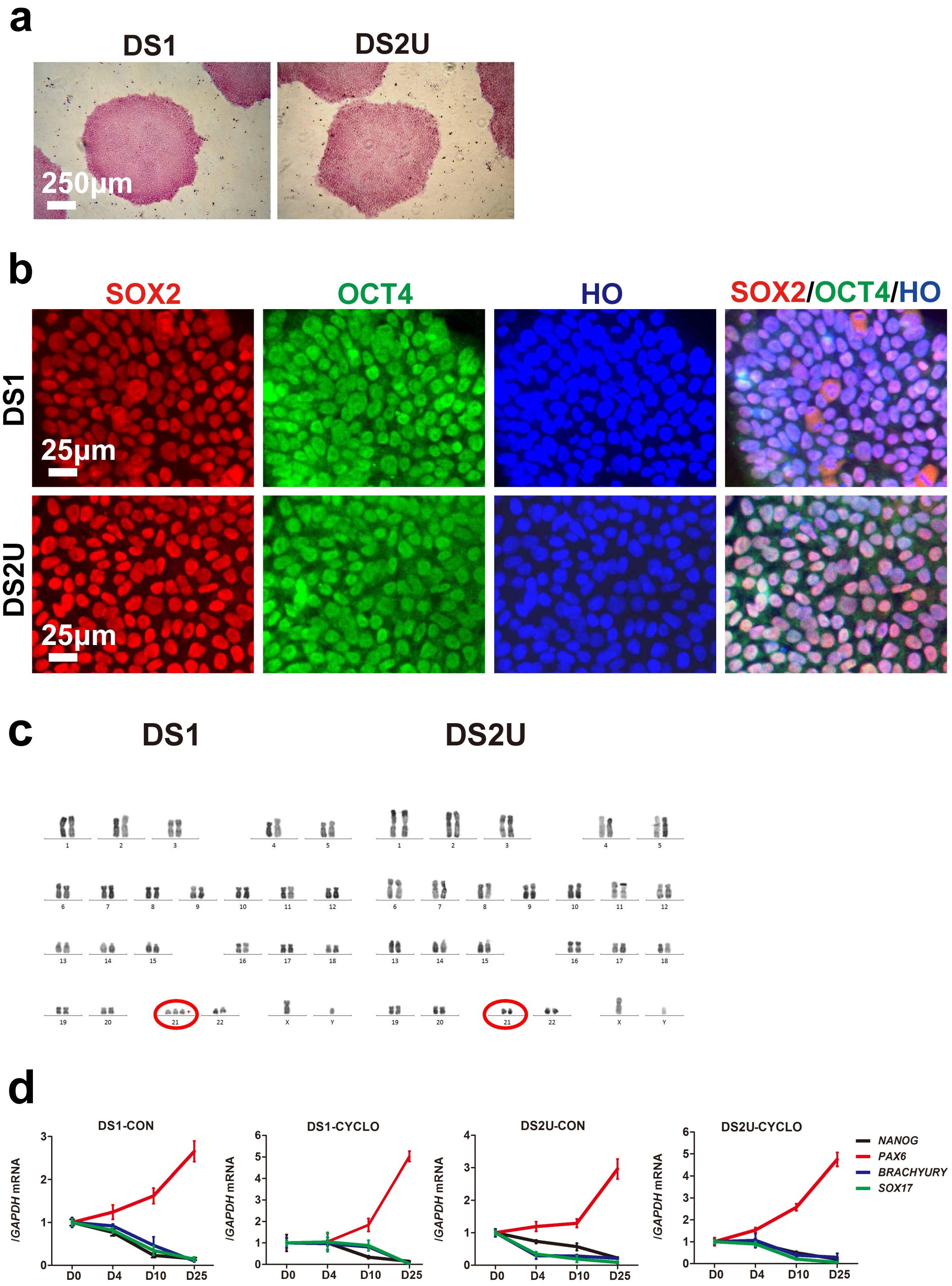

a
DS1
DS2U
250μm
b
SOX2
OCT4
HO
SOX2/OCT4/HO
DS1
DS2U
25μm
25μm
c
DS1
DS2U
d
DS1-CON
DS1-CYCLO
DS2U-CON
DS2U-CYCLO
/GAPDH mRNA
D0
D4
D10
D25
NANOG
PAX6
BRACHYURY
SOX17

Supplementary Table S1

Antibody	Isotype	Dilution	Source
TBR2	Rabbit IgG	1:200	Abcam
TBR1	Rabbit IgG	1:1000	Abcam
FOXG1	Rabbit IgG	1:1000	Abcam
MAP2	Mouse IgG	1:1000	Sigma-Aldrich
NESTIN	Goat IgG	1:1000	Santa Cruz Biotechnology
OTX2	Goat IgG	1:1000	R&D Systems
PAX6	Rabbit IgG	1:1000	Covance Research Products
SOX2	Rabbit IgG	1:1000	R&D Systems
SOX1	Rabbit IgG	1:1000	R&D Systems
TUJ-1	Rabbit IgG	1:2000	Covance Research Products
TUJ-1	Mouse IgG	1:2000	Sigma-Aldrich
synaptophysin	Rabbit IgG	1:200	Santa Cruz
DARPP32	Rabbit IgG	1:1000	Epitomics
GFAP	Rabbit IgG	1:1000	Dako
ISLET1	Mouse IgG	1:500	DSHB, Iowa City, IA
MEIS2	Goat IgG	1:500	Santa Cruz biotechnology
GAD67	Mouse IgG	1:1000	Chemicon and Millipore
GABA	Rabbit IgG	1:1000	Sigma-Aldrich
BNPI	Goat IgG	1:1000	Santa Cruz Biotechnology
vGLUT	Mouse IgG	1:1000	Synaptic System
GAPDH	Mouse IgG	1:4000	KangChen
KI67	Rabbit IgG	1:500	Life technology
CTIP2	Rat IgG	1:400	Abcam
SATB2	Mouse IgG	1:1000	Abcam

Supplementary Table S2

Gene	Forward Primer	Reverse Primer
SHH	AAGATCTCCAGAAACTCCGAGCGA	CGAGATGGCCAAAGCGTTCAACTT
GLI-1	GGCTGCAGTAAAGCCTTCAGCAAT	TGCAGCCAGGGAGCTTACATACAT
GLI-3	TTGCACAAAGGCCTACTCGAGACT	CTTGTTGCAACCTTCGTGCTCACA
PAX6	ACAGATCTACGCCAGCGACT	CATGGTGTCGTAGTGGATGC
EMX1	TTCAATGGGAGAGGGAGAGTGCTT	CCGTCAGCCTTTGTGAATGGTGTT
MASH1	GTCTCCCGGGGATTTTGTAT	TCTCCATCTTGGCAGAGCTT
DLX2	GCCTCAACAACGTCCCTTACT	TCACTATCCAATTTCAGGCTCA
NANOG	CAGCCCCGATTCTTCCACCAGTCCC	CGGAAGATTCCCAGTCGGGTTCACC
BRACHYURY	GCCCTCTCCCTCCCCTCCACGCACAG	CGGCGCCGTTGCTCACAGACCACAGG
SOX17	CGCTTTCATGGTGTGGGCTAAGGACG	TAGTTGGGGTGGTCCTGCATGTGCTG
GAPDH	TCGACAGTCAGCCGCATCTTCTTT	ACCAAATCCGTTGACTCCGACCTT
